# Gene network in pulmonary tuberculosis based on bioinformatic analysis

**DOI:** 10.1186/s12879-020-05335-6

**Published:** 2020-08-18

**Authors:** Lili Li, Jian Lv, Yuan He, Zhihua Wang

**Affiliations:** 1 Central Laboratory, Renmin Hospital of Wuhan University, 95 Zhangzhidong Rd. Wuchang District, Wuhan, 430060 China; 2 Department of Cardiology, Renmin Hospital of Wuhan University, 95 Zhangzhidong Rd. Wuchang District, Wuhan, 430060 China

**Keywords:** Pulmonary tuberculosis, *Mycobacterium tuberculosis*, Protein-protein interaction, Hub genes, Bioinformatic analysis

## Abstract

**Background:**

Pulmonary tuberculosis (PTB) is one of the serious infectious diseases worldwide; however, the gene network involved in the host response remain largely unclear.

**Methods:**

This study integrated two cohorts profile datasets GSE34608 and GSE83456 to elucidate the potential gene network and signaling pathways in PTB. Differentially expressed genes (DEGs) were obtained for Gene ontology (GO), Kyoto Encyclopedia of Genes and Genomes (KEGG) analysis using Metascape database. Protein-Protein Interaction (PPI) network of DEGs was constructed by the online database the Search Tool for the Retrieval of Interacting Genes (STRING). Modules were identified by the plug-in APP Molecular Complex Detection (MCODE) in Cytoscape. GO and KEGG pathway of Module 1 were further analyzed by STRING. Hub genes were selected for further expression validation in dataset GSE19439. The gene expression level was also investigated in the dataset GSE31348 to display the change pattern during the PTB treatment.

**Results:**

Totally, 180 shared DEGs were identified from two datasets. Gene function and KEGG pathway enrichment revealed that DEGs mainly enriched in defense response to other organism, response to bacterium, myeloid leukocyte activation, cytokine production, etc. Seven modules were clustered based on PPI network. Module 1 contained 35 genes related to cytokine associated functions, among which 14 genes, including chemokine receptors, interferon-induced proteins and Toll-like receptors, were identified as hub genes. Expression levels of the hub genes were validated with a third dataset GSE19439. The signature of this core gene network showed significant response to *Mycobacterium tuberculosis* (Mtb) infection, and correlated with the gene network pattern during anti-PTB therapy.

**Conclusions:**

Our study unveils the coordination of causal genes during PTB infection, and provides a promising gene panel for PTB diagnosis. As major regulators of the host immune response to Mtb infection, the 14 hub genes are also potential molecular targets for developing PTB drugs.

## Background

Pulmonary tuberculosis (PTB) is one of the serious infectious diseases with high mortality in the world. PTB is caused by various strains of mycobacteria with *Mycobacterium tuberculosis* (Mtb) being mostly observed in human. According to the World Health Organization (WHO) report, there were 10 million new cases of PTB disease and 1.5 million deaths worldwide in 2017 (WHO, 2018). It has been estimated that one third of the world’s population are infected with Mtb as latent infections, among which 5 to 10% would develop into active tuberculosis (TB) [1, 2]. Quick diagnostic and efficient treatment are of great importance to control the spread of PTB and reduce its mortality [3, 4]. Despite accumulating evidence on the mechanism of PTB, the molecular processes and the specific gene regulations in the progression of PTB remain to be explored.

Omics approaches, like genomics, transcriptomics, proteomics and metabolomics, are high-throughput methods that provide an opportunity to investigate the global gene expression changes in PTB [3]. Transcriptome profiling based on microarray or next-generation sequencing has been widely used for differentially expressed genes (DEGs) screening in human diseases. With the application of genechips, a large amount of data has been produced, most of which have been deposited in public databases. Integrating and re-analyzing these data provide valuable clues to advance our researches. In recently years, many microarray data profiling studies have been performed on PTB [5]. Through bioinformatic analysis, a number of DEGs and functional pathways have been identified [6]. However, these results are either inconsistent due to sample heterogeneity in individual studies, or limited by a single cohort study. So far, no reliable biomarkers are available for PTB diagnostics. Integrated bioinformatic analysis by combining these expression profiling data together would be a powerful approach to solve the disadvantages.

Here we analyzed two microarray datasets GSE34608 and GSE83456 from human whole blood samples including 53 health controls and 79 PTB samples. Multiple bioinformatics methods were employed to identify DEGs between the two datasets. Gene Ontology, pathway enrichment, Protein-Protein Interaction (PPI) network construction were performed to reveal the function of hub genes in PTB. Findings of this study might help to explore essential diagnostic signatures for PTB and shed a light on the molecular targets to treat PTB.

## Methods

### Gene expression microarray data acquisition

NCBI Gene Expression Omnibus database (GEO, http://www.ncbi.nlm.nih.gov/geo) is a public functional genomics database with high throughput gene expression sequencing data and microarrays data. Two gene expression datasets GSE34608 [7] and GSE83456 [6], were downloaded from GEO. GSE34608 contained 8 PTB samples and 18 control samples, which is based on GPL6480 platform (Agilent-014850 Whole Human Genome Microarray 4x44K G4112F). The GSE83456 dataset contained 45 PTB tissue samples and 61 control samples. It is based on GPL10558 platform (Illumina HumanHT-12 V4.0 expression beadchip). Another two datasets GSE19439 and GSE31348 were used for hub gene validation. GSE19439 contained 12 health and 13 PTB samples were used as validation dataset [8]. GSE19439 is based on GPL6947 platform (Illumina HumanHT-12 V3.0 expression beadchip). GSE31348 contained 27 subjects (135 samples) in five time point: diagnosis, treatment for 1, 2, 4 and 26 weeks, which is based on GPL570 platform (Affymetrix Human Genome U133 Plus 2.0 Array) [9].

### Identification of DEGs

Based on the microarray platform annotation, probe sets were converted into the corresponding gene symbol for the following analysis. Probe sets without corresponding gene symbols were removed. The DEGs between control and PTB samples were analyzed using limma (linear models for microarray data) package in R. |log2FC (fold change)| > 1 and adj. *P*-value < 0.05 were considered as statistically significant threshold for the DEGs selection of GSE34608. |log2FC| > 0.585 and adj. P-value < 0.05 were considered as statistically significant threshold for the DEGs selection of GSE83456. The co-existed DEGs were identified by drawing the venn diagram of DEGs of GSE34608 and GSE83456.

### KEGG and GO enrichment analyses of DEGs

Metascape (a gene annotation & analysis resource; http://metascape.org/) is online gene functional annotation tool to provide a comprehensive set of biological information of genes and proteins [10]. To understand the function of DEGs, Gene Oncology (GO) analysis, including biological process (BP), cellular components (CC), molecular function (MF), and KEGG pathway enrichment were performed using Metascape.

### PPI network construction and module analysis

In the present study, the PPI network was predicted using the Search Tool for the Retrieval of Interacting Genes (STRING; http://string-db.org) (version 11.0) online database [11]. The cut off value for STRING analysis is 0.04. Analyzing the functional interactions between proteins may provide insights into the biological mechanisms of action. PPI network were further visualized and analyzed with Cytoscape (version 3.4.0) plug-in APP Molecular Complex Detection (MCODE), which is used for clustering a given network based on topology. The most critical modules in the PPI network could be identified. The genes in top one module was displayed in this study. The hub gene selection criteria were as follows: MCODE scores > 10, degree > 20, neighborhood connectivity > 10.

### Hub genes analysis

The GO function, pathway, and protein domains of the top module were analyzed using STRING. The expression levels of hub genes were further validated in datasets GSE19439 and GSE31348.

## Results

### Identification of DEGs

**Fig. 1:**
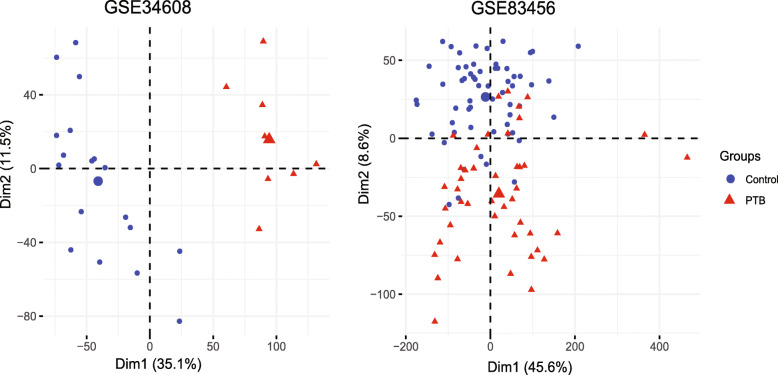
Principal component analysis (PCA) to discriminate the gene expression levels between control and pulmonary tuberculosis (PTB). **a** PCA plot of dataset GSE34608. **b** PCA plot of dataset GSE83456

**Fig. 2:**
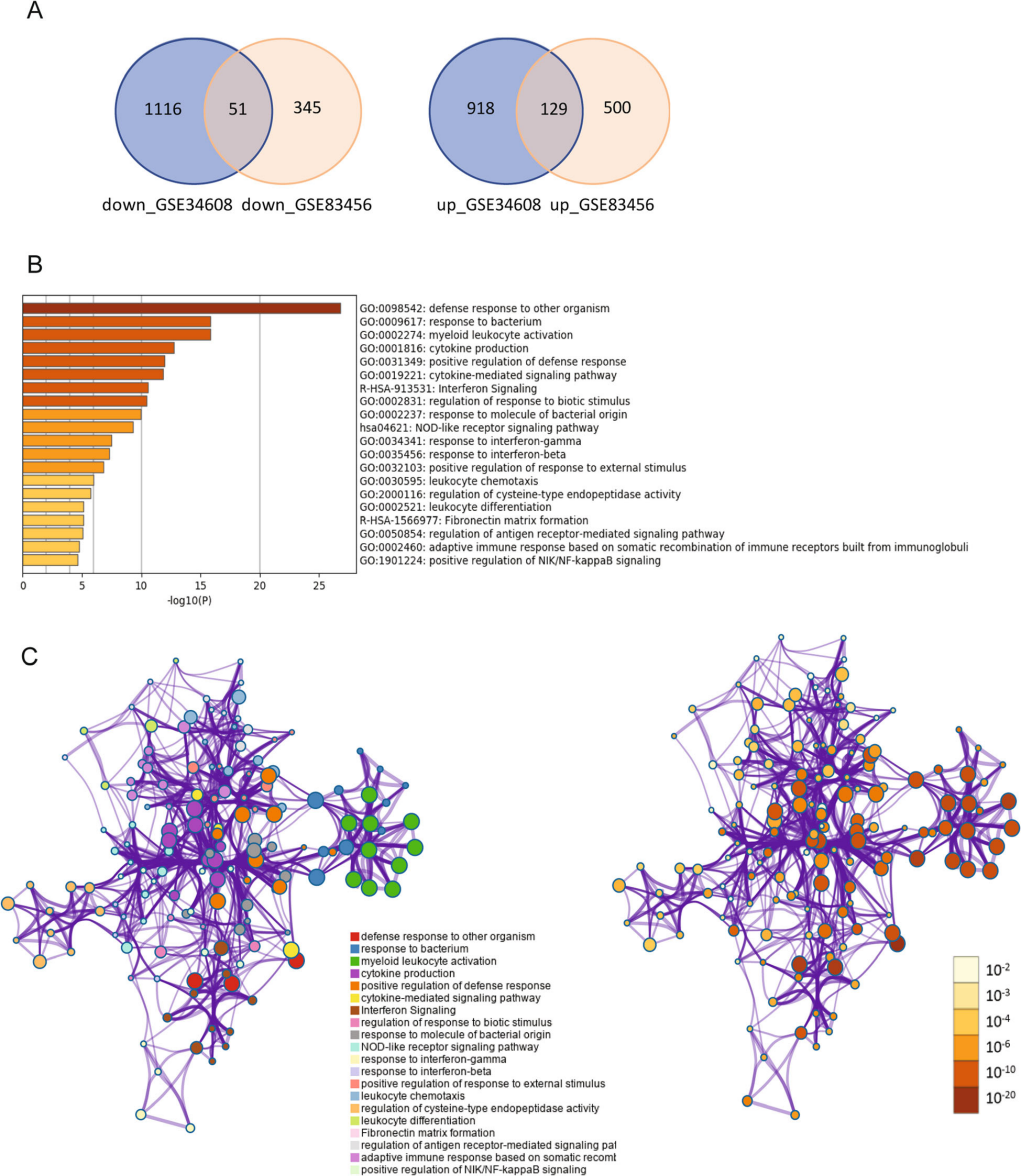
Selection and function of differentially expressed genes (DEGs). **a** Venn diagram of DEGs from the two datasets: GSE19439 and GSE34608. Totally 51 down-regulated and 129 up-regulated genes are shared between the two GSE datasets. **b** and **c** Functional annotation of DEGs using Metascape. The top 20 terms are displayed as bar plot based on *P* value (log10 scale) (**b**), and the network plot (**c**)

Gene expression profile of GSE34608 and GSE83456 were downloaded from GEO database. The microarray data GSE34608 contains 18 control and 8 PTB patients. The GSE83456 data contains 61 control samples and 45 PTB samples. PCA plots of both datasets indicated the distinction expression of control and PTB samples (Fig. 1a and b). 2214 and 1025 DEGs were identified from GSE34608 and GSE83456 datasets, respectively (Fig. 2a). Venn diagram demonstrated that, among the 180 shared DEGs, 51 genes were down-regulated and 129 genes were upregulated in both datasets (Fig. 2a).

### GO enrichment and KEGG pathway analyses

Candidate DEGs functional Gene Ontology (GO) and pathway enrichment analyses were performed with Metascape. The results showed that DEGs were significantly enriched in defense response to other organism, response to bacterium, myeloid leukocyte activation, cytokine production, positive regulation of defense responses, cytokine-mediated signaling pathway, interferon signaling, etc. (Fig. 2b).

The subset of representative terms of gene function analysis were converted into a network layout in Metascape, as shown in Fig. 2c. Based on gene function analysis, all the significant terms were hierarchically clustered into a tree based on Kappa-statistical similarities. Each term is represented by a circle node, where its size is proportional to the number of input genes fall into that term. The color represents its cluster identity (Fig. 2c). Terms with a kappa score > 0.3 are linked by an edge. The statistically significant range of the node is marked by color range (Fig. 2c).

### PPI network construction and module analysis

**Fig. 3:**
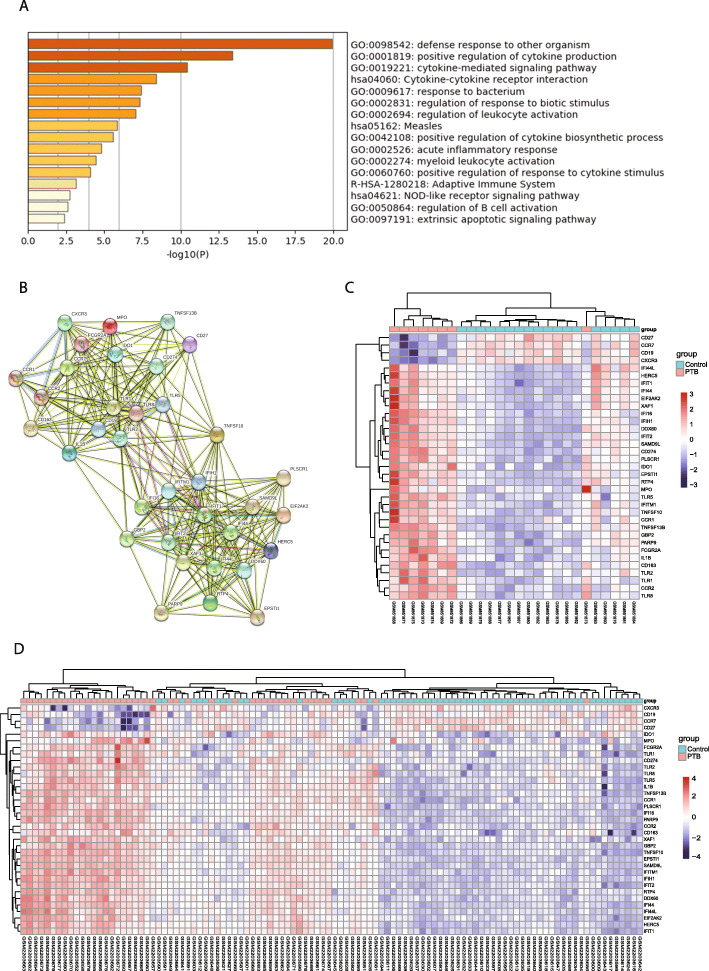
Functional analysis of 35 genes in Module 1. **a** GO analysis reveals that genes functionally related to defense response to other organism underlie PTB infection. **b** Network of Module 1 genes constructed by STRING. **c** Heatmap showing the gene expression of module 1 in individuals from dataset GSE34608. **d** Heatmap showing the gene expression of module 1 in individuals from dataset GSE83456. Each row represents a gene, and each column represents a sample

The PPI network of 180 DEGs was constructed using the STRING online database, and further analyzed using app MCODE in Cytoscape software. Totally, seven modules were identified shown in Table 1. Module 1 from the PPI network complex contained 35 genes, indicating the core functional gene panel. GO analysis of 35 genes showed that their functions are related to defense response and cytokine related pathway (Fig. 3a). PPI network of module 1 was redrawn by STRING (Fig. 3b). The expression level of 35 genes in dataset GSE34608 were shown in Fig. 3c. Genes CD27, CCR7, CD19, and CXCR3 were significantly down-regulated in PTB samples, where other genes were upregulated. This result was consistent with the gene expression levels in GSE83456 (Fig. 3d). Furthermore, function analysis from STRING database were shown in Table 2. GO function was significantly related to defense response and immune system response (biological process function), chemokine binding and chemokine receptor activity (molecular function), and external side of plasma membrane and cell surface (cellular component). KEGG and Reactome pathways indicated that these 35 genes were involved in cytokine-cytokine receptor interaction, Toll-like receptor signaling pathway, immune system, and cytokine signaling in immune system. TIR domain, leucine rich repeat, and chemokine receptor family were the three important features revealed by PFAM and INTERPRO protein domains analysis (Table 2).

**Table 1:** Seven modules were identified by MCODE based on the 180 DEGs

Module	Gene symbol
Module 1	TLR2 IL1B TLR8 TLR1 IFIH1 TLR5 IFIT1 CD19 IFIT2 CCR7 MPO CXCR3 IFI44 DDX60 FCGR2A CD163 IFI44L GBP2 TNFSF10 CD274 CCR2 XAF1 IFI16 IFITM1 IDO1 HERC5 SAMD9L EIF2AK2 RTP4 CCR1 CD27 PLSCR1 TNFSF13B PARP9 EPSTI1
Module 2	CXCL10 GBP5 ELANE AIM2 LCN2 DEFA4 HP NLRC4 MMP8 LTF TCN1 HPSE
Module 3	STAT1 LCK FAS
Module 4	CEACAM1 GPR84 BST1
Module 5	FAM26F SPPL2A USP25
Module 6	DBP TLE2 AES
Module 7	CAMP CEACAM8 S100A12 RNASE3

**Table 2:** Function analysis of the 35 genes in module 1

Term	Description	Count	FDR
**Biological Process (GO)**			
GO:0006952	Defense response	28 of 1234	2.39E-24
GO:0002376	Immune system process	31 of 2370	1.2E-21
GO:0051707	Response to other organism	22 of 835	4.56E-19
GO:0006955	Immune response	25 of 1560	9.48E-18
GO:0002682	Regulation of immune system process	24 of 1391	1.52E-17
**Molecular Function (GO)**			
GO:0019956	Chemokine binding	4 of 22	0.0000233
GO:0004950	Chemokine receptor activity	4 of 27	0.0000243
GO:0019957	C-C chemokine binding	3 of 11	0.000088
GO:0016493	C-C chemokine receptor activity	3 of 13	0.00011
GO:0003725	Double-stranded RNA binding	4 of 70	0.00028
**Cellular Component (GO)**			
GO:0009897	External side of plasma membrane	9 of 223	2.93E-08
GO:0009986	Cell surface	11 of 690	0.0000015
GO:0035354	Toll-like receptor 1-Toll-like receptor 2 protein complex	2 of 2	0.00064
GO:0005887	Integral component of plasma membrane	11 of 1564	0.0017
GO:0044459	Plasma membrane part	13 of 2651	0.0083
**KEGG Pathways**			
hsa04060	Cytokine-cytokine receptor interaction	8 of 263	0.00000133
hsa04620	Toll-like receptor signaling pathway	5 of 102	0.0000409
hsa05162	Measles	5 of 133	0.0000955
hsa05168	Herpes simplex infection	5 of 181	0.00031
hsa05134	Legionellosis	3 of 54	0.0019
**Reactome Pathways**			
HSA-168256	Immune system	22 of 1925	3.84E-12
HSA-1280215	Cytokine signaling in immune system	12 of 654	5.29E-08
HSA-913531	Interferon signaling	7 of 189	0.00000192
HSA-168249	Innate immune system	12 of 1012	0.0000032
HSA-909733	Interferon alpha/beta signaling	5 of 66	0.0000039
**PFAM Protein Domains**			
PF01582	TIR domain	4 of 22	0.0000057
PF13855	Leucine rich repeat	4 of 187	0.0051
PF13676	TIR domain	2 of 11	0.0051
PF13306	Leucine rich repeats (6 copies)	3 of 88	0.0051
PF01463	Leucine rich repeat C-terminal domain	2 of 12	0.0051
**INTERPRO Protein Domains and Features**			
IPR035897	TIR domain superfamily	4 of 26	0.00000766
IPR000355	Chemokine receptor family	4 of 21	0.00000766
IPR000157	TIR domain	4 of 22	0.00000766
IPR024644	Interferon-induced protein 44 family	2 of 2	0.00031
IPR000483	Cysteine-rich flanking region, C-terminal	4 of 83	0.00031

### Hub genes analysis

**Fig. 4:**
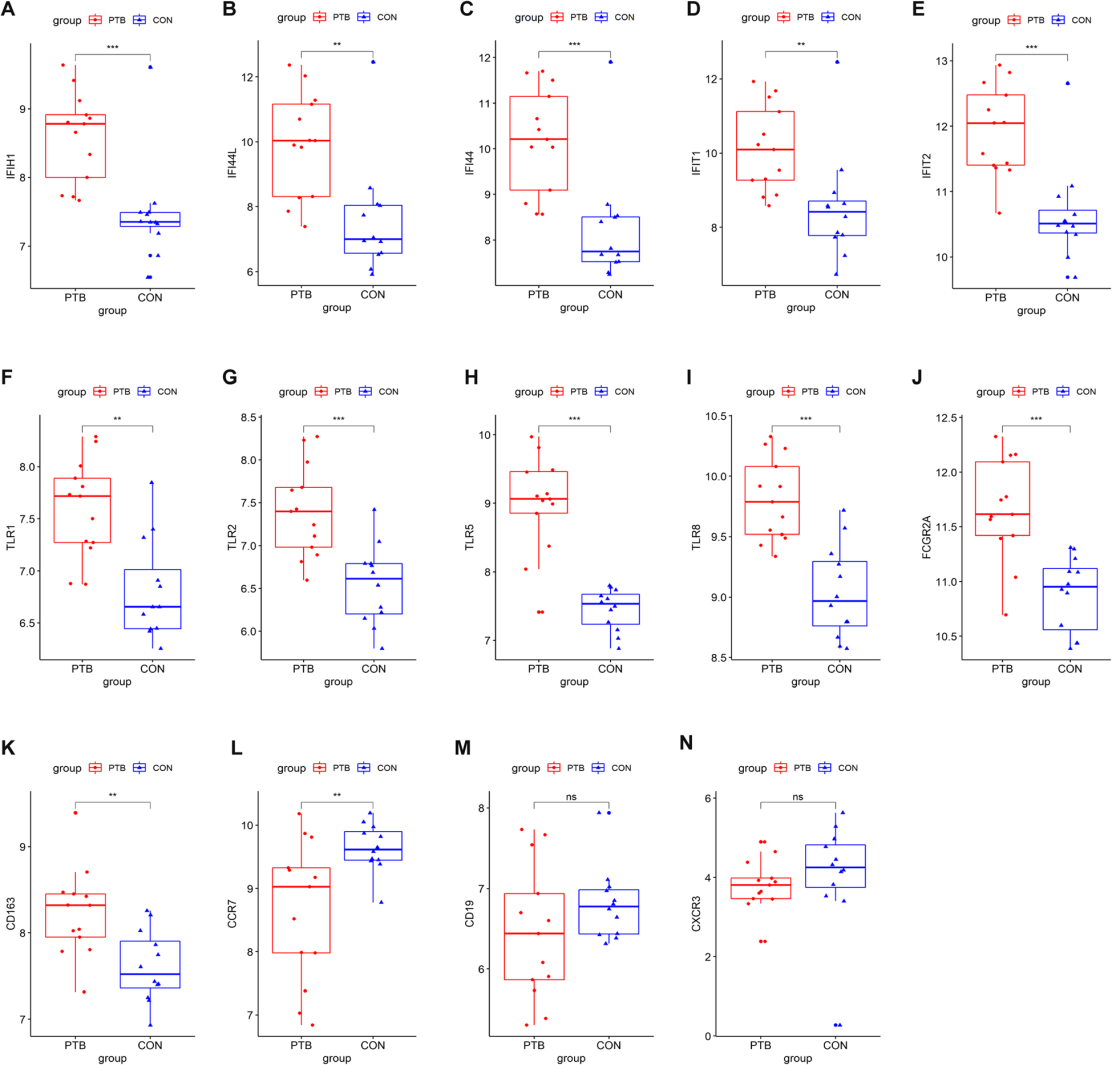
Expression validation of the hub genes in dataset GSE19439. **a-n** Demonstrate the expression of IFIH1, IFI44L, IFI44, IFITI, IFIT2, TLR1, TLR2, TLR5, TLR8, FCCGR2A, CD163, CCR7, CD19, CXCR3 during PTB infection, respectively. **P value < 0.01; ***P value < 0.001; ns indicates not significant

A total of 14 genes were selected as hub genes based on criteria MCODE (scores > 10, degree > 20, neighborhood connectivity > 10) in Table 3. All the hub genes were belonging to the module 1. These hub genes were significantly associated with Toll-like receptors, interferon-induce proteins, and chemokine receptors (Table 3). Among them, two genes were upregulated, whereas others were downregulated (Fig. 3c). The expression levels were further validated in dataset GSE19439 (Fig. 4). The expression levels were also significantly different between health and PTB patients, except gene CD19 and CXCR3 (Fig. 4).

**Table 3:** The functions of 14 hub genes

Gene	Score^a^	Full name	Function^b^
CD163	12	Scavenger receptor cysteine-rich type 1 protein M130	Acute phase-regulated receptor involved in clearance and endocytosis of hemoglobin/haptoglobin complexes by macrophages and may thereby protect tissues from free hemoglobin-mediated oxidative damage.
TLR5	11.02941	Toll-like receptor 5	Participates in the innate immune response to microbial agents.
IFIT1	10.82353	Interferon-induced protein with tetratricopeptide repeats 1	Interferon-induced antiviral RNA-binding protein that specifically binds single-stranded RNA bearing a 5′-triphosphate group (PPP-RNA), thereby acting as a sensor of viral single- stranded RNAs and inhibiting expression of viral messenger RNAs.
IFIT2	10.82353	Interferon-induced protein with tetratricopeptide repeats 2	IFN-induced antiviral protein which inhibits expression of viral messenger RNAs lacking 2′-O-methylation of the 5′ cap.
CCR7	10.7451	C-C chemokine receptor type 7	Receptor for the MIP-3-beta chemokine. Belongs to the G-protein coupled receptor 1 family.
CXCR3	11.2	C-X-C chemokine receptor type 3	Isoform 1- Receptor for the C-X-C chemokine CXCL9, CXCL10 and CXCL11 and mediates the proliferation, survival and angiogenic activity of human mesangial cells (HMC) through a heterotrimeric G-protein signaling pathway.
TLR1	10.7451	Toll-like receptor 1	Participates in the innate immune response to microbial agents. Specifically recognizes diacylated and triacylated lipopeptides.
FCGR2A	10.63736	Low affinity immunoglobulin gamma Fc region receptor II-a	Binds to the Fc region of immunoglobulins gamma.
CD19	10.7451	B-lymphocyte antigen CD19	Assembles with the antigen receptor of B-lymphocytes in order to decrease the threshold for antigen receptor-dependent stimulation.
IFIH1	10.82353	Interferon-induced helicase C domain-containing protein 1	Innate immune receptor which acts as a cytoplasmic sensor of viral nucleic acids and plays a major role in sensing viral infection and in the activation of a cascade of antiviral responses including the induction of type I interferons and proinflammatory cytokines.
IFI44L	10.82353	Interferon-induced protein 44-like	Exhibits a low antiviral activity against hepatitis C virus.
TLR8	10.7451	Toll-like receptor 8	Key component of innate and adaptive immunity.
IFI44	10.82353	Interferon-induced protein 44	This protein aggregates to form microtubular structures.
TLR2	10.7451	Toll-like receptor 2	Cooperates with LY96 to mediate the innate immune response to bacterial lipoproteins and other microbial cell wall components.

Score^a^: indicated MCODE score. Function^b^: obtained from NCBI (https://www.ncbi.nlm.nih.gov/)

### Gene expression level detection during PTB treatment

**Fig. 5:**
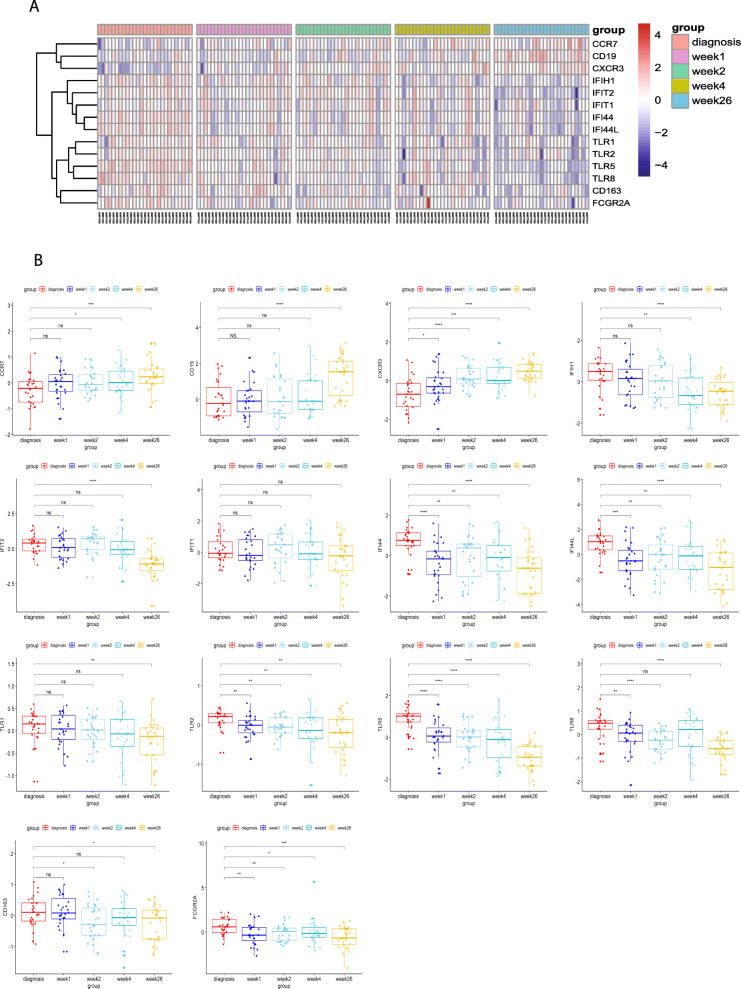
The expression levels of hub genes during PTB treatment in five time points in dataset GSE31348. Heatmap (**a**) and box plot (**b**) of hub genes in dataset GSE31348. The central of rectangle indicates the medium. The ends of the whiskers represent the first quartile to the third quartile (the interquartile range or IQR). The ends of central line show the minimum or maximum value. *P value < 0.05; **P value < 0.01; ***P value < 0.001; ns indicates not significant

To figure out the expression level changes during PTB treatment process, dataset GSE31348 were used to evaluate the change level. GSE31348 contained the 27 PTB patients, including 135 samples from 5 time points. Heatmap showed that the expression level of genes related with the functions (Fig. 5a). The expression level of CCR7, CD19, and CXCR3 were significantly increased, whereas the expression level of Interferon-induced proteins, Toll-like receptors were decreased during the treatment (Fig. 5a). Among these 14 genes, the expression level of CXCR3 were significantly increased, and TLR2 and TLR5 were significantly decreased during the PTB treatment (Fig. 5b). These three genes might have a potential to evaluate PTB as a gene panel.

## Discussion

In this study, we analyzed two GEO datasets GSE34608 and GSE83456 to identify hub genes related to PTB disease. Totally, 180 DEGs were identified by combining these two GEO datasets (Fig. 2). With STRING protein-protein interaction data, 14 hub genes were identified (Figs. 3 and 4). The function of these 14 hub genes were chemokine receptors, interferon-induced proteins and Toll-like receptors (Tables 2 and 3). The signature of hub genes are significantly correlated with PTB infection as well as anti-PTB therapy (Fig. 5).

Our study reveals the core genes in response to Mtb infection. The gene expression profile in PTB patients conforms to the common inflammatory responses upon viral and bacterial infections [12]. Chemokine receptors, interferon-induced proteins, and Toll-like receptors were involved in this core response profiling, and significantly changed following successful treatment.

Chemokines play a major role in the host response to Mtb infection as they contribute to the formation and maintenance of quiescent granulomas and the establishment of the TB granuloma. High concentrations of cytokines and chemokines are required for early protection against Mtb infections, but may also be involved in the over-response of host immune system [13]. Plasma cytokines can serve as biomarkers for the disease severity, and function to relieve the mycobacterial burden in PTB disease [14]. Consistent with our analyses, a previous study has reported that PTB patients displayed significantly elevated levels of CCL1, CCL3, CXCL1, CXCL10 and CXCL11 which were significantly reduced following successful treatment [14].

Type I interferon response pathway is a well-established pathway crucial for the defense against viral pathogens, but it could also be detrimental upon infection with mycobacteria [15]. Although the signaling axis through this pathway is identical regardless of the type of infection, the outcome is substantially different, suggesting that the type I IFNs and the related IFN-inducible genes are able to create a favorable or unfavorable intracellular milieu to promote or disrupt the survival of invading pathogens [16]. Changes in mRNA levels of IFIT1, IFIT2, CXCR3 and CD163 have been validated by qPCR in previous studies [17–19]. Moreover, Kim et al. [20] confirmed that five genes (IFN-γ, TNF-α, IL-2R, CXCL9, and CXCL10) could be used for the detection of Mtb infection, including active PTB disease and LTB with sensitivity of each gene above 80%. The gene panel revealed in this study provides a more comprehensive network for selecting diagnostic biomarkers. However, it needs to be further tested in other infectious diseases to figure out the transcriptional signature specific to PTB disease or shared with other types of infections.

The essential role of Toll-like receptors against mycobacterial infection has been revealed in vivo. Toll-like receptors play key roles in innate and adaptive immunity against Mtb, and are involved in the recognition of conserved microbial structures, leading to activation of an inflammatory response. Previous study showed that TLR3 and TLR5 were upregulated at 24, 48 and 72 h post-infection in A549 pulmonary epithelial cells treated with Mtb [21], and the expression of neutrophil TLR2 is also increased in PTB patients [22]. Whole blood from patients had increased mRNA levels of TLR1 and TLR2 [23]. TLR2-deficient mice showed increased subsequent progression to PTB disease, the rapid death and higher Mtb burden [24]. TLR2 may function as a regulator of inflammation, and its absence exacerbated the detrimental inflammatory response. TLR1 rs5743551 and rs5743618 polymorphisms significantly increased under the Mtb infection in 203 PTB patients, compared to 203 healthy subjects [24]. TLR8 polymorphisms rs3764879 and rs3764880 have also been reported to cause differential sensitivity to Mtb infection by specific strains [25].

Gene function can be regulated at multiple levels. Integrated multi-omics analysis provides a better approach to understand the comprehensive biological processes in human diseases. By integrating transcriptomics, proteomics and metabolomics, Zhao et al. [26] revealed the molecular link between lipid metabolism and inflammatory response in chronic obstructive pulmonary disease (COPD) treated with a Chinese medicine Bufei Jianpi Formula in a rat model. Similar strategies were also applied to study the Laser Printer-Emitted Nanoparticles (PEPs) inhalation exposure-induced disease risks to identify metabolite biomarkers [27]. In Mtb research, metabolomics has been applied to investigate the metabolic traits in Mtb species [28, 29]. However, omics study other than transcriptomics in the host system under Mtb infection is limited. Changes in host proteome and metabolome after Mtb infection need to be further investigated to provide a more detailed landscape to understand the molecular mechanism of PTB disease.

## Conclusion

In summary, we construct a refined gene network representing the transcriptome signature in response to Mtb infection and its treatment. The identified 14 hub genes are promising biomarkers for developing transcriptome-based PTB diagnostic or prognostic tests. As major regulators of the host immune response to Mtb infection, these genes are also potential molecular targets for developing drugs to treat PTB.

## Data Availability

Datasets including GSE34608, GSE83456, GSE19439 and GSE31348 were downloaded from NCBI Gene Expression Omnibus database (GEO, http://www.ncbi.nlm.nih.gov/geo).
